# Sprouty2—a Novel Therapeutic Target in the Nervous System?

**DOI:** 10.1007/s12035-018-1338-8

**Published:** 2018-09-17

**Authors:** Barbara Hausott, Lars Klimaschewski

**Affiliations:** 1 Department of Anatomy, Histology and Embyrology, Division of Neuroanatomy, Medical University Innsbruck, Müllerstrasse 59, 6020 Innsbruck, Austria; 2 Division for Neuroanatomy, Medical University of Innsbruck, Müllerstrasse 59, 6020 Innsbruck, Austria

**Keywords:** Glioma, Epilepsy, Stroke, Nerve lesion, Degeneration, Regeneration, ERK

## Abstract

Clinical trials applying growth factors to alleviate symptoms of patients with neurological disorders have largely been unsuccessful in the past. As an alternative approach, growth factor receptors or components of their signal transduction machinery may be targeted directly. In recent years, the search for intracellular signaling integrator downstream of receptor tyrosine kinases provided valuable novel substrates. Among them are the Sprouty proteins which mainly act as inhibitors of growth factor-dependent neuronal and glial signaling pathways. In this review, we summarize the role of Sprouties in the lesioned central and peripheral nervous system with particular reference to Sprouty2 that is upregulated in various experimental models of neuronal degeneration and regeneration. Increased synthesis under pathological conditions makes Sprouty2 an attractive pharmacological target to enhance intracellular signaling activities, notably the ERK pathway, in affected neurons or activated astrocytes. Interestingly, high Sprouty2 levels are also found in malignant glioma cells. We recently demonstrated that abrogating Sprouty2 function strongly inhibits intracranial tumor growth and leads to significantly prolonged survival of glioblastoma bearing mice by induction of ERK-dependent DNA replication stress. On the contrary, knockdown of Sprouty proteins increases proliferation of activated astrocytes and, consequently, reduces secondary brain damage in neuronal lesion models such as kainic acid-induced epilepsy or endothelin-induced ischemia. Furthermore, downregulation of Sprouty2 improves nerve regeneration in the lesioned peripheral nervous system. Taken together, targeting Sprouties as intracellular inhibitors of the ERK pathway holds great promise for the treatment of various neurological disorders including gliomas. Since the protein lacks enzymatic activities, it will be difficult to develop chemical compounds capable to directly and specifically modulate Sprouty functions. However, interfering with Sprouty expression by gene therapy or siRNA treatment provides a realistic approach to evaluate the therapeutic potential of indirectly stimulating ERK activities in neurological disease.

## Sprouties as Regulators of RTK Signaling in Neurons and Glia

**Fig. 1:**
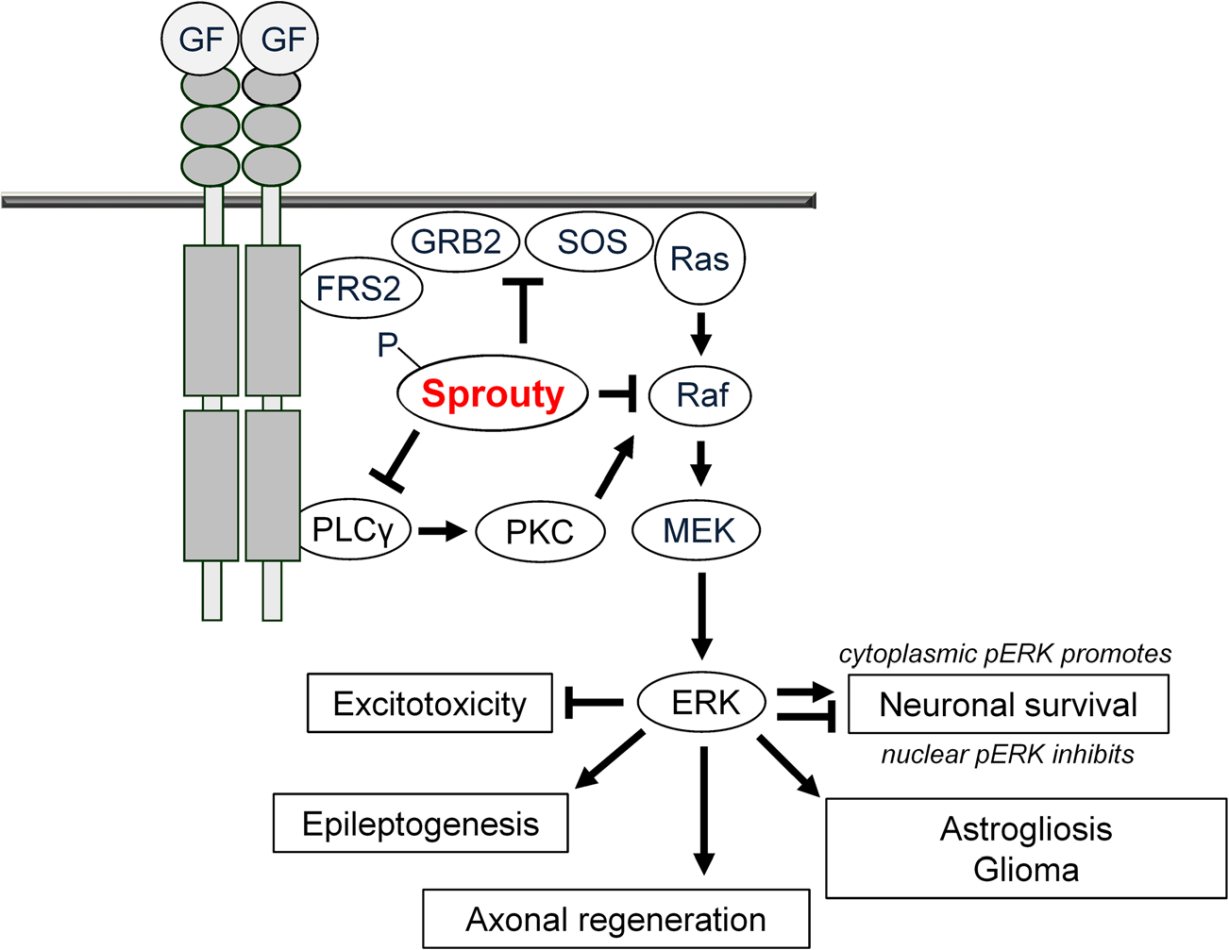
Sprouties have originally been described as inhibitors of FGF-induced tracheal branching in drosophila. Spry1, Spry2, and Spry4 but not Spry3 are induced transcriptionally and limit the duration and intensity mainly of ERK phosphorylation in response to growth factor (GF) stimulation (with the exception of EGF signaling). Receptor dimerization and autophosphorylation attracts proteins containing Src homology 2 (SH2) or phosphotyrosine binding (PTB) domains including adaptor proteins like FRS2 and GRB2. Son of sevenless (SOS) is then recruited to the plasma membrane and catalyzes the conversion of inactive Ras-GDP to active Ras-GTP that in turn recruits Raf to the plasma membrane. Raf family members will activate MEK1/2 followed by phosphorylation of ERK1/2 which acts on a large variety of targets. These regulate neuronal survival (depending on duration and location), glial proliferation, axonal regeneration, and neuronal activity

Sprouty (Spry) proteins are inhibitors of receptor tyrosine kinase (RTK) signaling [1, 2]. They were originally discovered as fibroblast growth factor (FGF) antagonists [3, 4] but later found to act on signaling mechanisms induced by activation of other RTKs as well [5–7]. RTK-dependent signaling pathways provide a variety of targets for the treatment of neurological and neuropsychiatric disorders in which neurotrophins and other growth factors are released [8, 9] (Fig. 1). However, over recent years, it became clear that RTKs cannot be sufficiently activated by growth factors or receptor agonists in the adult and aging brain to exert significant neuroprotective or neurorestorative effects. The reasons for this may involve receptor downregulation and truncation, among others [10].

Four functionally conserved Spry isoforms exist. Spry1, Spry2, and Spry4 represent the major isoforms, whereas Spry3 is detected at low levels in the brain only [4]. Spry1, Spry2, and Spry4 are all detected in the neopallial cortex, cranial flexure, and cerebellum [11]. In postnatal mouse brains, Spry2 and Spry4 represent the major CNS isoforms in neuronal and glial cells of the cerebellum, cortex, and hippocampus [12, 13]. Transfecting dominant-negative Spry2 results in an anterior shift of the posterior border of the tectum during brain development, whereas overexpression of Spry2 induces a fate change of the presumptive metencephalon to the mesencephalon [14]. Spry2 overexpression also blocks neurite formation in immature cerebellar granule neurons, while inhibition of Spry2 by a dominant-negative mutant or siRNA promotes neuritogenesis [12] and axon outgrowth by embryonic hippocampal as well as sensory neuron cultures [15, 16]. Hence, Sprouties play a key role in neuronal differentiation and proliferation [17] and in regulating progenitor identity in the ventricular zone [18]. Interestingly, Spry1 and Spry2 can also act in a non-cell autonomous fashion by restricting FGF signaling and allowing Wnt expression in peripheral progenitor cells. Wnt ligands are then released and bind to parasympathetic neuronal precursor cells resulting in the formation of parasympathetic ganglia [19]. The importance of Sprouties during development is further underscored by the observation that their complete deletion significantly perturbs cell differentiation and/or organ morphogenesis, such as in the gastrointestinal tract [20] or in the inner ear where homozygous Spry2 deficiency disrupts the cytoarchitecture of the organ of Corti [21].

## Sprouties in the Lesioned Central Nervous System

**Fig. 2:**
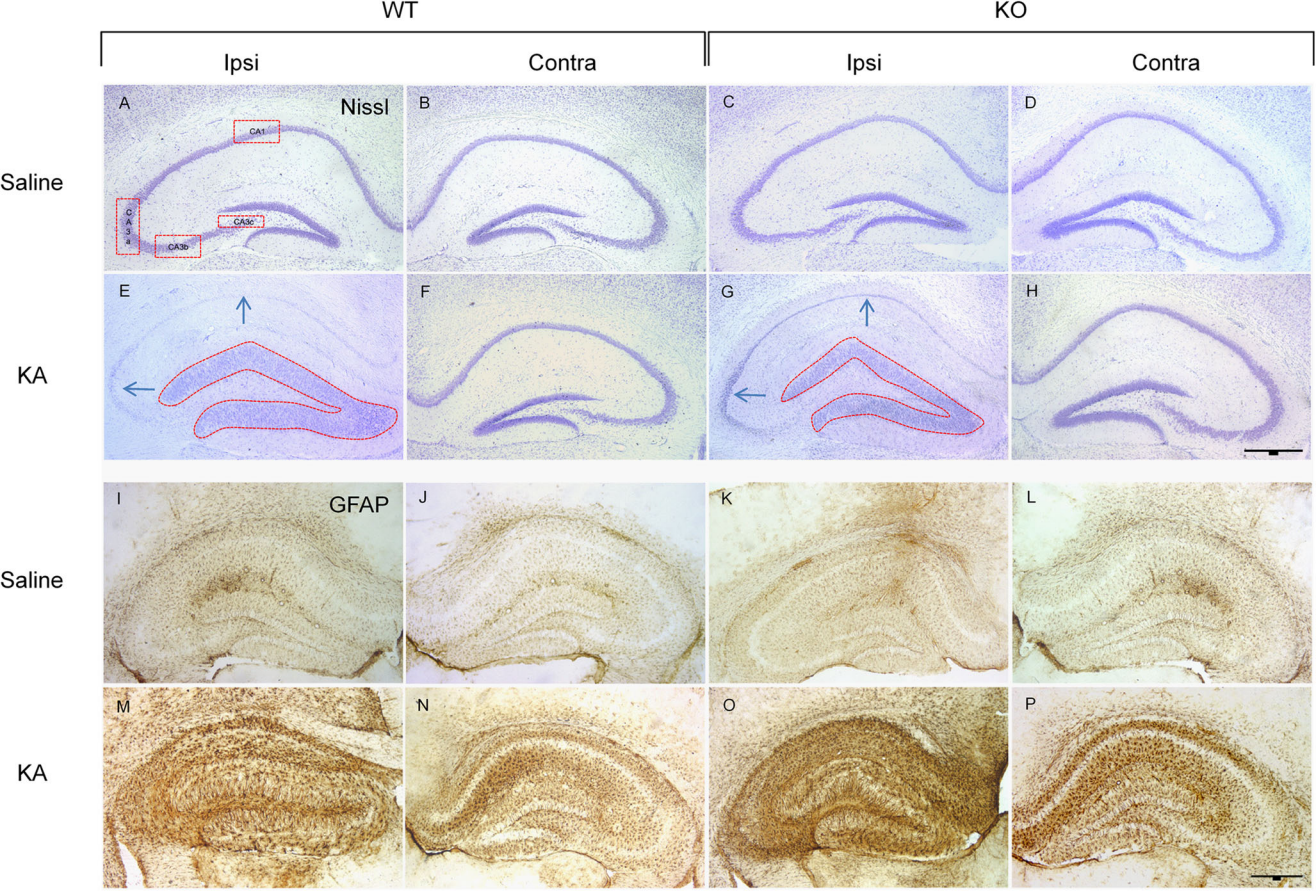
**a**–**h** Nissl staining followed by stereological analysis of neuronal loss and granule cell dispersion 3 weeks after unilateral intrahippocampal injection of saline or kainic acid (KA). Images of 30-μm sections of the dorsal hippocampus near the injection site are shown (1.8 mm caudal to bregma). KA-induced cell death and granule cell dispersion are clearly visible in WT (**a**, **b**, **e**, **f**) and in Spry2/4^+/−^ mice (**c**, **d**, **g**, **h**). Red boxes in panel **a** indicate the hippocampal subregions analyzed in this study, boxes in **e** and **g** the areas measured for quantification of granule cell dispersion and arrows indicating affected CA1/CA3 pyramidal layers. **i**–**p** Glial fibrillary acidic protein (GFAP) immunohistochemistry in sections neighboring **a**–**h**. As compared to saline injection (**i**–**l**), prominent KA-induced reactive astrocytosis is detected in the ipsilateral and contralateral hippocampus of both genotypes (**m**–**p**) and further enhanced in mice with reduced Spry2/4 levels (**o**, **p**). Taken from [22]

With regard to the adult CNS, we demonstrated that Sprouty proteins play a role in limiting secondary brain damage in a mouse model of kainate-induced epileptogenesis [22]. Mesial temporal lobe epilepsy is one of the most common types of epilepsy characterized by recurrent spontaneous seizures that often result in hippocampal sclerosis and granule cell dispersion. Spry2/4 heterozygote double-knockout mice exhibit stronger ERK activation in the hippocampal CA3 pyramidal cell layer and hilar region. Following induction of epilepsy, neuronal migration of dentate granule cells (i.e., dispersion) is diminished in these animals and neuronal degeneration reduced in CA1 and CA3 principal neuron layers (Fig. 2). The number of reactive astrocytes markedly increases in lesioned areas of Spry2/4^+/−^ mice as compared to that of wildtype animals. Hence, although the seizure threshold is reduced in naive Spry2/4 heterozygous knockout mice, neurodegeneration and granule cell dispersion are mitigated following kainic acid-induced hippocampal lesions.

Spry proteins are efficiently downregulated by injection of specific siRNAs into rodent brain tissues [23]. In this study, we demonstrated that siRNA-mediated Spry2/4 reduction diminishes the size of the lesion 3 weeks after endothelin-induced vasoconstriction (a mouse model for human stroke), most likely due to a more pronounced reactive astrocytic response in the peri-infarct area. Apart from cell death in the core region, ischemia induces a series of morphological and biochemical alterations in the surrounding penumbra which are counteracted by the early increase in astrogliosis.

Astrocytes perform both detrimental and beneficial functions for neuronal survival during the acute phase of ischemia. Inflammatory astrocytic responses may exacerbate the ischemic lesion, but astrocytes also limit the extension of the lesion via anti-excitotoxic effects and release of growth factors. Therefore, astrocytes constitute important therapeutic targets to improve the functional outcome following stroke particularly in penumbral tissue [24].

Almost every brain lesion is accompanied by reactive astrogliosis and involves morphological and biochemical changes of pre-existing astrocytes as well as formation of new astrocytes from stem cells [25]. Ablating reactive proliferating astrocytes following traumatic brain injury markedly enhances neural damage and promotes leukocyte infiltration into the affected area [26]. Thus, the prevalent view of reactive astrogliosis as an inhibitor of recovery and regeneration has changed into a more optimistic prospect emphasizing the beneficial functions of ERK-induced astrogliosis and scar formation, even as promotors of axonal regeneration, for example, following spinal cord injury [27].

Dis-inhibiting the ERK pathway by decreasing Spry2/4 levels does not only enhance reactive astrogliosis but appears to be an important mechanism to block glioma growth as well. Recently, we corroborated an oncogenic role of Spry2 in glial brain tumors [28]. Spry2 expression is upregulated in patients with highly malignant gliomas (GBM) which correlates with reduced overall survival supporting previous findings that gliomas with high expression of Spry isoforms (Spry1, Spry2, and Spry4) and low expression of NF1 and PTEN are associated with poor prognosis as compared to tumors with a reversed expression pattern [29]. Knockdown of Spry2 significantly impairs proliferation of GBM cells in vitro and in vivo. EGF-induced ERK and AKT activation increases concomitantly resulting in signaling stress with premature S-phase entry. Consistent with these findings, DNA damage response and cytotoxicity are enhanced.

## Sprouties in the Lesioned Peripheral Nervous System

Peripheral nerves are provided with the ability to regenerate after injury. Although regeneration of the peripheral nervous system is more successful than regeneration of the central nervous system, functional recovery after peripheral nerve injury is still highly limited [9]. Therefore, improvement of long-distance axon growth is required for fast regeneration of axons into target tissues to avoid atrophy in the absence of innervation. Interference with Spry2 may provide a novel approach to promote axon elongation in lesioned peripheral nerves via enhanced ERK signaling.

Spry2 is regulated post-transcriptionally by miR-21 in response to a peripheral nerve transection [30]. Upregulation of miR-21 was detected in the dorsal root ganglion (DRG) 2 days after axotomy, and this increase was sustained up to 28 days after injury. Although Spry2 mRNA levels are not altered in response to a sciatic nerve lesion [16], the persistent upregulation of miR-21 after axotomy results in decreased Spry2 protein levels during nerve regeneration. Heterozygous Spry2 knockout mice recover faster in motor testing paradigms indicating that Spry2 is involved in long-distance regeneration [31]. In fact, an improvement in behavioral motor tests, higher numbers of myelinated fibers in the regenerating sciatic nerve, higher densities of motor endplates in hind limb muscles, and increased levels of GAP-43 mRNA, a downstream target of ERK signaling, are observed in Spry2^+/−^ mice when compared to wild-type littermates.

**Fig. 3:**
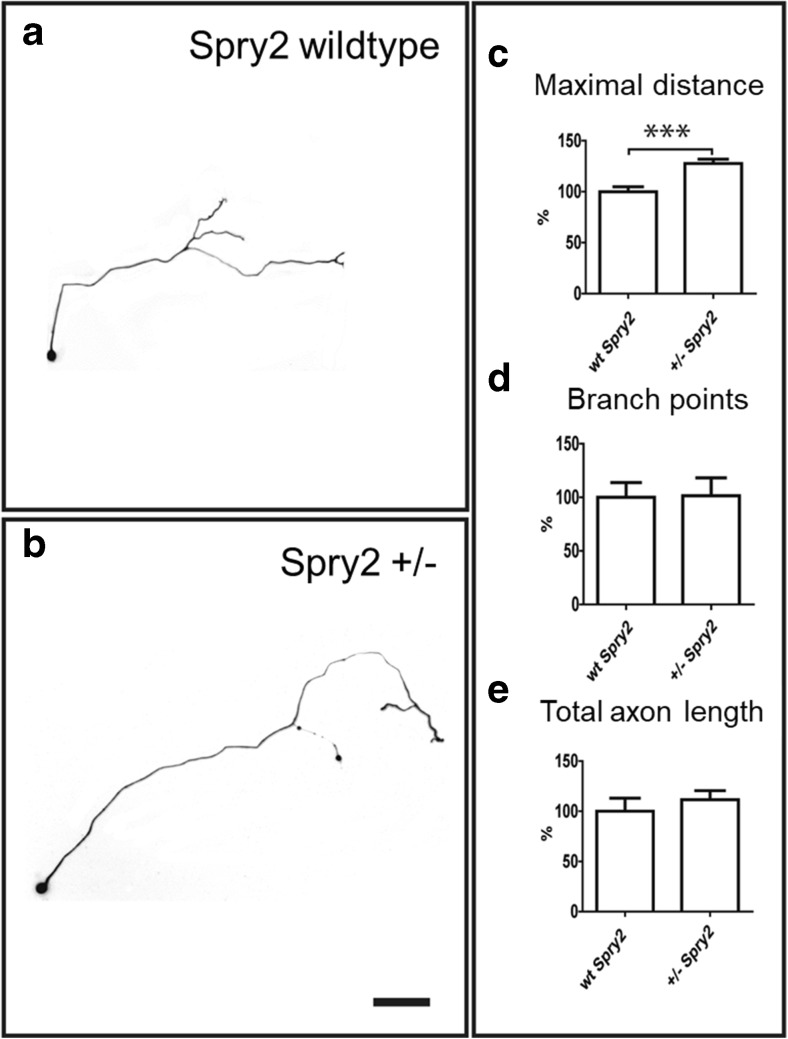
Representative examples of neuronal morphologies of sympathetic superior cervical ganglion neurons stained for the tubulin marker Tuj-1. Inverted fluorescence images are shown to document the axonal tree (**a**, **b**). In cultures obtained from Spry2^+/−^ mice, the length of the longest axon (maximal distance) is significantly increased (**c**). In contrast, there is no change in axon branching (**d**) or total axon length (**e**). Taken from [31]

Adult primary sensory or sympathetic neurons dissociated from heterozygous Spry2^+/−^ mice reveal stronger ERK activation and enhanced axon outgrowth (Fig. 3), while homozygous Spry2^−/−^ neurons exhibit a branching phenotype. Axon outgrowth and elongation of Spry2^+/−^ neurons are further enhanced by FGF-2 and NGF treatment. By contrast, Spry2^−/−^ neurons do not exhibit significantly increased axon outgrowth in response to growth factor treatment [31]. Quantitative RT-PCR reveals a 2.6-fold increase of tropomyosin receptor kinase A (TrkA) mRNA in the DRG from Spry2^−/−^ but not from Spry2^+/−^ mice (unpublished observation). These results require further investigation, but it is well known that activation of TrkA induces axon branching of adult DRG neurons [32]. Taken together, we propose that partial downregulation, but not complete silencing, of Spry2 will be beneficial to promote axon elongation and long-distance nerve regeneration without induction of axon branching in the PNS.

Cellular aspects of peripheral nerve regeneration, such as axon branching and elongation as well as Schwann cell proliferation, are strongly influenced by neuronal growth factors via activation of various RTK-dependent signaling mechanisms including the ERK and phosphatidylinositol 3-kinase (PI3K) pathways [33, 34]. In developing sensory neurons, activation of ERK induces axonal elongation, whereas active AKT increases axon caliber and branching [35]. A comparison of the effects of FGF-2 and NGF on ERK and AKT signaling demonstrates that FGF-2 induces stronger ERK than AKT activation, while NGF results in both, ERK and AKT phosphorylation, to a similar extent [16]. FGF-2 but not NGF increases elongative axon growth in pre-lesioned neuron cultures [36] corroborating the significance of ERK over AKT signaling in promotion of long-distance axon regeneration induced by peripheral nerve injury. Therefore, interference with Sprouties to specifically enhance ERK but not AKT signaling in neurons appears advantageous over growth factor treatment which will activate both, ERK and AKT signaling, downstream of RTKs.

## Molecular Mechanisms of Spry2 Action

**Fig. 4:**
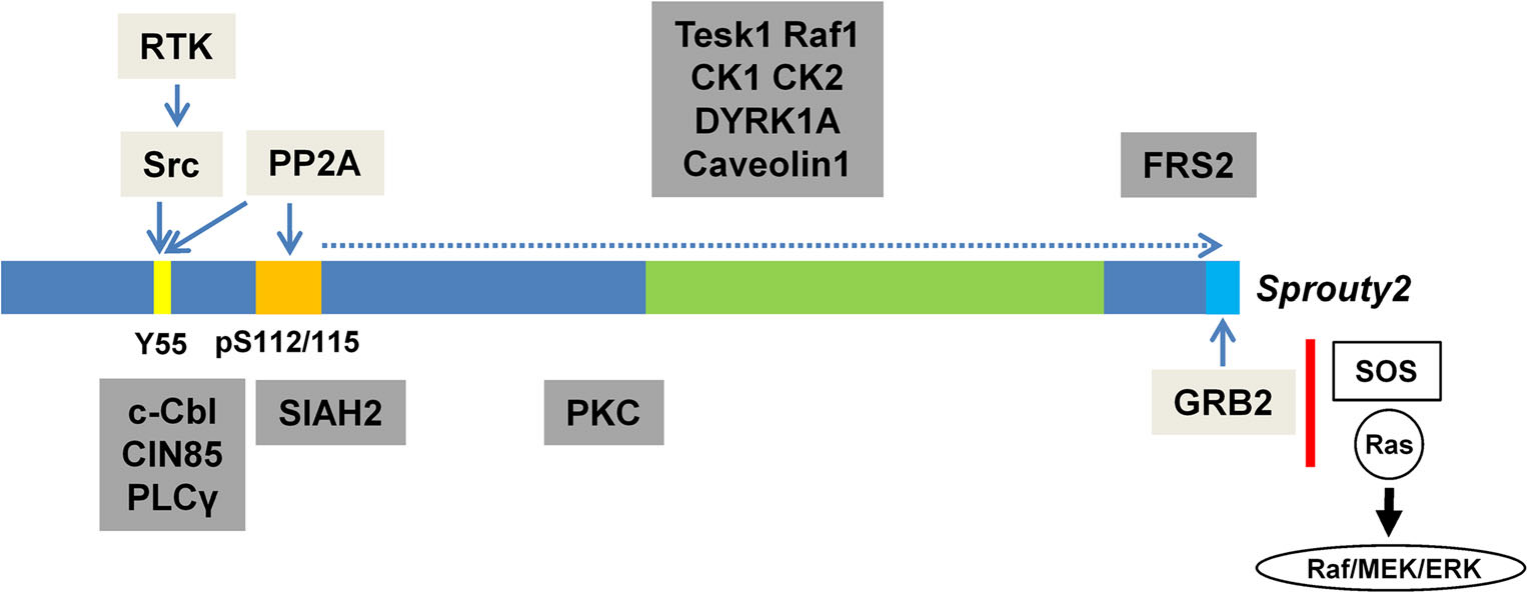
Activation of Spry2 (conserved cysteine-rich region in green) in response to receptor tyrosine kinase (RTK) stimulation is induced by Src kinase (Y55 phosphorylation, Y53 for Spry4) followed by phosphatase recruitment (PP2A) which dephosphorylates serines 112 and 115. This results in conformational change at the C-terminal proline-rich binding site for GRB2 (light blue, present in Spry2 only) that now becomes accessible and prevents interaction with SOS required for downstream ERK activation (interacting proteins indicated in light gray boxes). Other binding partners and covalent modifiers (listed in dark gray boxes) have been demonstrated to interact mainly with human Spry2. Based on sequence similarities and experimental evidence, however, many of them also bind Spry1 and Spry4 [39, 40]. Spry4 has been shown to inhibit the ERK pathway predominantly by interaction with SOS1, and all Sprouties may form functionally active hetero- and homo-oligomers through their C-terminal domains [41]. Src, non-receptor kinase; C-Cbl, Casitas b-lineage lymphoma ubiquitin E3 ligase; CIN85, Cbl-interacting protein of 85 kD; PLCγ, phospholipase Cγ; SIAH2, seven in absentia homolog ubiquitin E3 ligase; PP2A, protein phosphatase 2A; PKC, protein kinase C; Tesk1, testicular protein kinase 1; CK, casein kinase; DYRK1A, dual-specificity tyrosine-phosphorylation-regulated kinase 1A

Spry1, Spry2, and Spry4 are upregulated transcriptionally by growth factor stimulation and normally inhibit the pathways by which they are induced (hence their characterization as negative feedback inhibitors - with the exception of EGF signaling). Following RTK activation, they translocate from the cytosol to the cell membrane where they become anchored through palmitoylation and phosphorylated at tyrosine residues [37, 38]. Spry2 activation requires phosphorylation at the essential Tyr55 residue via c-Src kinase (Fig. 4). Spry4 is not phosphorylated in response to RTK stimulation; however, the Tyr53 residue is necessary for its inhibitory activity [42]. While Spry1 and Spry2 are strongly phosphorylated following RTK activation, the phosphorylation of Spry4 is weak [39]. Spry2 is considerably more inhibitory than Spry1 or Spry4 which correlates with the binding of Spry2 to GRB2 via a C-terminal proline-rich sequence that is found exclusively in Spry2 [43].

The assigned major role of Spry1, Spry2, and Spry4 is the inhibition of the ERK pathway (Fig. 1). Spry2 binds the adaptor protein GRB2 and thereby prevents ERK activation upstream of Ras [44]. Moreover, Spry2/4 interacts with Raf downstream of Ras [45, 46]. Thus, Spry2 interferes with the ERK pathway upstream and downstream of Ras. Among the different Spry isoforms, Spry2 reveals a stronger inhibitory effect on ERK activation than Spry1 or Spry4 [45]. Spry3 exerts only a weak effect on ERK inhibition in Xenopus, whereas no effect of Spry3 on ERK signaling is observed in mammalian cells [47]. However, in case of constitutively activated Ras, Spry2 has no inhibitory effect on the ERK pathway. Hence, constitutively active Ras can circumvent the function of Spry2 as an inhibitor of ERK in tumor cells [6]. In addition to the ERK pathway, Spry1 and Spry2 inhibit activation of PLCγ and Spry4 blocks activation of PKC [48, 49].

The effect of Spry2 on the AKT pathway seems to be dependent on the cell type and the growth factor involved. The AKT pathway is not impaired by Spry2 in some studies [6, 45], whereas evidence was provided for an inhibitory role of Spry2 on AKT signaling by enhancing the activity of phosphatase and tensin homolog deleted on chromosome 10 (PTEN) [50]. We found that in adult sensory neurons, downregulation of Spry2 leads to activation of Ras and pERK in response to FGF-2, whereas phosphorylation of pAKT and p38 remains unchanged [16, 31]. Interestingly, we observed activated ERK primarily in the cytoplasm, but not in the nucleus of Spry2-deficient peripheral neurons. Although speculative at the moment, this may be caused by different activities of the ERK1/2 inactivating MAP kinase phosphatases [51]. Whereas MKP1 is located in the nucleus, MKP3 is a cytoplasmic enzyme. Therefore, specific targeting of MKP3 may result in similar effects as interference with Spry2 to increase cytoplasmic ERK activation.

All Spry proteins exhibit a highly conserved C-terminal cysteine-rich region and a variable N-terminal region, both containing various phosphorylation sites (Fig. 4). Kinases like dual-specificity tyrosine-phosphorylated and regulated kinase 1A (DYRK1A), testicular protein kinase 1 (TESK1), and MAPK-interacting kinase 1 (Mnk1) and phosphatases like PTEN, protein phosphatase 2 A (PP2A), Src homology-2-containing phosphotyrosine phosphatase (SHP2), and protein tyrosine phosphatase 1B (PTP1B) regulate the biological activity of Sprouties [39, 52]. The ubiquitin ligase casitas b-lineage lymphoma (c-Cbl) and seven in absentia homolog 2 (SIAH2) interact with the N-terminus for control of ubiquitination and degradation of Spry2. In non-neuronal cells expressing EGF receptors, overexpression of Sprouties (Spry1, Spry2) increases ERK signaling by binding and sequestering c-Cbl, thereby impeding EGFR ubiquitylation and degradation [7, 53]. In addition, Spry may target Cbl to other proteins for ubiquitination or functions as adaptor protein for Cbl via its scaffolding but not via its E3 ligase function [39].

## Clinical Implications

The results obtained so far provide a novel therapeutic avenue to enhance neuroprotective and pro-regenerative signaling by interfering with Sprouty proteins in several neurological diseases. We now much better understand inhibition of intracellular signaling pathways in neurons and astrocytes under normal and pathological conditions. It is to be expected that efficient siRNA treatments and viral gene transfer will be available for human brain therapy in the future. In fact, gene replacement therapy promotes survival of patients with spinal muscular atrophy following a single intravenous infusion of adeno-associated virus containing DNA coding for *SMN1* [54]. Lentiviral vectors are particularly useful for in vivo applications, because of their efficiency in gene delivery and excellent safety profile. Also, in contrast to retroviral vectors, lentiviral vectors do not depend on active division of the cell to be transduced. They have a large cloning capacity, high transduction efficiency, and sustained transgene expression and can be engineered to restrict gene expression to particular cell subtypes. In our lab, we currently produce LVs that mediate cell-type-specific transduction in the central nervous system [55]. They combine specific promoters, a tetracycline-dependent self-regulating (Tet) system and an indirect miRT detargeting strategy to avoid transgene expression in unwanted cells. Lentiviral vectors have been successfully applied recently to promote axonal regeneration in CNS lesion models [56, 57].

In conclusion, the detailed knowledge of the pathogenic mechanisms of neurodegeneration, axon regeneration, and astrogliosis did not lead to causal therapeutic consequences in the past. Hence, downregulation of Spry2 will have the potential to become a promising tool as a symptomatic treatment for delaying neuronal degeneration, enhancing nerve regeneration, and even inhibiting brain tumor growth via stimulation of RTK-dependent signaling pathways in neurons and glial cells.
